# Study of Interactions between Amlodipine and Quercetin on Human Serum Albumin: Spectroscopic and Modeling Approaches

**DOI:** 10.3390/molecules24030487

**Published:** 2019-01-30

**Authors:** Zuzana Vaneková, Lukáš Hubčík, José Luis Toca-Herrera, Paul Georg Furtműller, Jindra Valentová, Pavel Mučaji, Milan Nagy

**Affiliations:** 1 Department of Pharmacognosy and Botany, Faculty of Pharmacy, Comenius University in Bratislava, 83232 Bratislava, Slovakia; mucaji@fpharm.uniba.sk (P.M.); nagy@fpharm.uniba.sk (M.N.); 2 Department of Physical Chemistry of Drugs, Faculty of Pharmacy, Comenius University in Bratislava, 83232 Bratislava, Slovakia; hubcik@fpharm.uniba.sk; 3 Department of Nanobiotechnology, University of Natural Resources and Life Sciences, 1190 Vienna, Austria; jose.toca-herrera@boku.ac.at; 4 Department of Chemistry, University of Natural Resources and Life Sciences, 1190 Vienna, Austria; paul.furtmueller@boku.ac.at; 5 Department of Chemical Theory of Drugs, Faculty of Pharmacy, Comenius University in Bratislava, 83232 Bratislava, Slovakia; valentova@fpharm.uniba.sk

**Keywords:** human serum albumin, amlodipine, quercetin, fluorescence, circular dichroism, FT-IR, molecular modeling

## Abstract

The aim of this study was to analyze the binding interactions between a common antihypertensive drug (amlodipine besylate—AML) and the widely distributed plant flavonoid quercetin (Q), in the presence of human serum albumin (HSA). Fluorescence analysis was implemented to investigate the effect of ligands on albumin intrinsic fluorescence and to define the binding and quenching properties. Further methods, such as circular dichroism and FT-IR, were used to obtain more details. The data show that both of these compounds bind to Sudlow’s Site 1 on HSA and that there exists a competitive interaction between them. Q is able to displace AML from its binding site and the presence of AML makes it easier for Q to bind. AML binds with the lower affinity and if the binding site is already occupied by Q, it binds to the secondary binding site inside the same hydrophobic pocket of Sudlow’s Site 1, with exactly the same affinity. Experimental data were complemented with molecular docking studies. The obtained results provide useful information about possible pharmacokinetic interactions upon simultaneous co-administration of the food/dietary supplement and the antihypertensive drug.

## 1. Introduction

Human serum albumin (HSA) is the most prevalent protein in human plasma, constituting ~60% of the total plasma protein content. Its structure and purpose have been studied in great detail and it offers a wide variety of applications in research as well as in clinical medicine [1].

HSA and its ability to bind and transport a wide range of molecules (e.g., drugs, metabolites, fatty acids, etc.) play a key role in drug distribution. The most well-known binding sites are Sudlow’s Site 1 (located at subdomain IIA and containing a tryptophan residue) and Site 2 (located on subdomain IIIA) [2,3,4].

HSA in the presence of multiple ligands creates a complex system, where several molecules may or may not compete to bind to the same binding site. Since only the free fraction of the total amount of drug is responsible for the therapeutic effect, these competitive displacement interactions should be considered when administering multiple drugs [5].

The novel aspect of pharmacokinetic interaction which has arisen in the recent years is the displacement of drugs from their binding sites by plant metabolites. Phenolic compounds like flavonoids or tannins naturally occur in all plant materials, whether their use is dietary (the daily intake of fresh fruit and vegetables) or medicinal (herbal teas and supplements). Several studies have explored these interactions between selected drugs and various plant polyphenols, such as warfarin with quercetin [6] and multiple other flavone, flavonol, and flavanone aglycones [7]; nifedipine with rutin and baicalin [8]; ticagrelor with quercetin; rutin with baicalin [9]; propranolol with quercetin [10]; and gliclazide with quercetin [11]. All of these studies came to a common conclusion that flavonoids bind with high affinity to HSA and are able to alter the binding of the other molecule.

**Figure 1:**
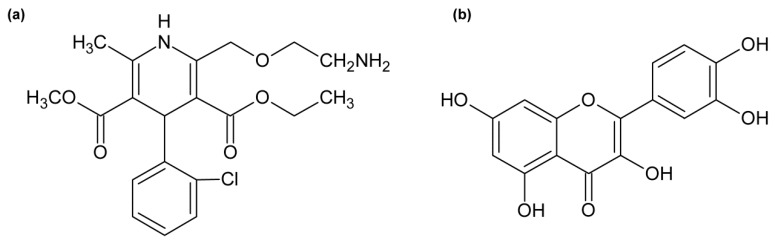
Structures of (**a**) amlodipine and (**b**) quercetin.

Amlodipine (AML; Figure 1a) is a long-acting calcium channel blocker belonging to the dihydropyridine derivatives group. Members of this group selectively inhibit the transmembrane influx of calcium ions into vascular smooth muscle which causes a peripheral vasodilatation. AML is widely used for the treatment of hypertension in the form of a besylate salt, often in combination with other types of antihypertensives [12]. In vitro studies have shown that approximately 93% of circulating amlodipine is bound to plasma proteins [13]. Other studies extensively proved that AML binds to the Site 1 on HSA [14,15,16,17]. Its (*S*)-enantiomer binds to a higher extent than the (*R*)-enantiomer [18].

Quercetin (Q; Figure 1b) is one of the most commonly occurring flavonoids in any plant material and one of the most abundant dietary flavonoids with an average daily consumption of 25–50 mg [19]. It is known for its strong antioxidant, anti-inflammatory, and venoprotective properties. After consumption, quercetin undergoes an extensive glucuronide conjugation in liver. Four of the five hydroxyl groups can be conjugated with glucuronic acid (with the exception of 5-hydroxyl group) and the most common metabolite is quercetin-3-glucuronide [19,20]. Quercetin and its metabolites strongly bind to HSA (99.4% for quercetin) and the main binding site is Sudlow’s Site 1 [6,9,10,11,21,22]. 

The aim of this study was to investigate a possibility of binding interactions between AML and Q in the presence of HSA which, to the best of our knowledge, has not yet been studied. The competitive binding was investigated first by fluorescence spectroscopy. A more detailed view on conformational alterations of HSA was obtained by implementing circular dichroism and FT-IR techniques. Molecular docking studies were used to complement the experimental data. 

## 2. Results

### 2.1. Fluorescence Quenching and Enhancement

Fluorescence spectroscopy is one of the most widely used methods for study of protein–ligand interactions due to its convenience, high reliability, and sensitivity. Intrinsic fluorescence of proteins is caused by aromatic amino acid residues, namely tryptophan (Trp), tyrosine (Tyr), and phenylalanine (Phe). Trp fluorescence is usually dominant in the protein emission spectrum, with excitation maximum wavelength at 295 nm and emission maximum wavelength at 350 nm. Tyrosine has a quantum yield similar to tryptophan, but its emission spectrum is more narrowly distributed on the wavelength scale, occurs at shorter wavelengths and is generally quenched in the protein matrix. Fluorescence spectra are highly sensitive to changes on the environment around amino acid residues: excited-state reactions, molecular rearrangements, changes in solvent polarity, energy transfer, ground-state complex formation, and collisional quenching. These result in changes of the fluorescence intensity (quenching or enhancement) and shifts in maximum wavelengths [23].

**Figure 2:**
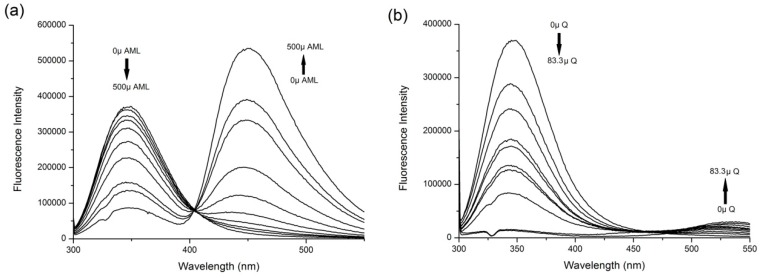
The fluorescence emission spectra of (**a**) amlodipine besylate (AML) + human serum albumin (HSA) and (**b**) Q + HSA systems at excitation λ = 295 nm. Conditions: T = 310.15 K, pH = 7.4. The HSA concentration was 5 μM; AML concentrations were 0, 3.9, 7.8, 15.6, 31.25, 62.5, 125, 250, 333.3, and 500 μM; Q concentrations were 0, 1.95, 3.9, 7.8, 10.4, 15.6, 20.8, 31.25, 41.6, 62.5 and 83.3 μM.

HSA structure consists of 585 amino acids with 18 Tyr residues and one Trp residue (located at position 214), which is conveniently placed inside the hydrophobic pocket of the Sudlow’s Site 1. HSA in solution presented a strong fluorescence emission with a peak maximum at 347 nm when excitation wavelength was 295 nm. The studied drugs had no intrinsic fluorescence at these wavelengths. However, increasing concentrations of AML and Q in the HSA solution result in large-scale quenching of HSA intrinsic fluorescence (Figure 2), which indicates that both drugs bind into the close proximity of Trp^214^ in Site 1. There is also a blue shift in the Q + HSA system spectra, indicating that the polarity around Trp residue decreased. The AML + HSA system does not show any considerable shift in maximum wavelengths. 

**Figure 3:**
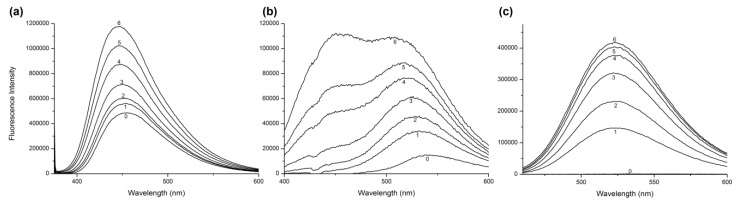
The fluorescence emission spectra of ligand + HSA systems for (**a**) AML at λ_EX_ = 370 nm, (**b**) Q at λ_EX_ = 375 nm, and (**c**) Q at λ_EX_ = 450 nm. In all experiments, the ligand concentration was 7.5 μM and HSA concentrations were 0 (line 0), 1.87 (line 1), 3.75 (line 2), 7.5 (line 3), 15 (line 4), 20 (line 5), and 30 (line 6) μM. Conditions: T = 310.15 K, pH = 7.4.

Both studied drugs contain conjugated double bond systems in their structures (Figure 1). AML shows observable fluorescence emission at λ_EX_ = 370 nm and λ_EM_ = 450 nm and Q shows weak fluorescence emission at λ_EX_ = 375 nm and λ_EM_ = 540 nm. HSA does not show any intrinsic fluorescence at these wavelengths. Therefore, we were also able to observe changes in ligand fluorescence emission. Upon addition of HSA increasing concentrations into ligand solutions, we observed significant increase of the ligand fluorescence for both AML and Q (Figure 3). 

Quercetin fluorescence shows an unusual dual behavior, as described by Sengupta & Sengupta [22]. At λ_EX_ = 375 nm we observed an enhancement of intrinsic fluorescence with a significant blue shift at λ_EM_ = 525 nm, and in samples with higher concentrations of HSA, a second peak with emission maximum at λ_EM_ = 450 nm appears, which is characteristic of its parent compound, 3-hydroxyflavone (Figure 3b). If we use a different excitation wavelength, λ_EX_ = 450 nm, which is a selective excitation wavelength for Q + HSA ground-state complex, a single emission band appears with a maximum at λ_EM_ = 525 nm (Figure 3c). 

**Figure 4:**
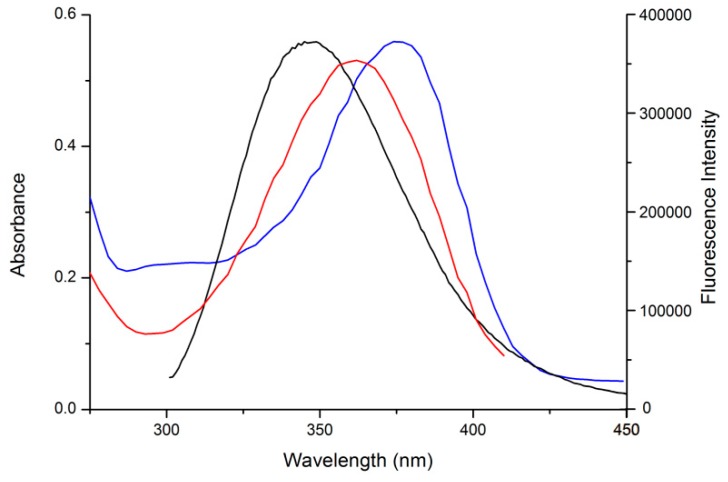
Comparison of absorption spectra of AML (**red line**), Q (**blue line**), and fluorescence emission spectra of HSA (**black line**).

Fluorescence resonance energy transfer (FRET) occurs when the donor (in this case Trp^214^ of HSA) and acceptor (in this case AML or Q) exist in a close proximity and the donor emission spectrum overlaps with the absorption spectrum of the acceptor [23]. Figure 4 shows that both studied compounds provide an unusually strong spectral overlap. In Figure 2, both secondary mixtures (AML + HSA and Q + HSA) show bands at λ_EM_ = 450 nm for AML and λ_EM_ = 530 nm for Q, respectively. This behavior further supports that both compounds bind to the close proximity of Trp^214^. 

In order to find out if there was any interaction between the two studied compounds and the binding site on HSA, we performed an analysis of ternary mixtures, [(Q + HSA) + AML] and [(AML + HSA) + Q], respectively. The comparison of quenching curves in the binary and ternary systems provides general information concerning the change of affinity of one drug to serum albumin in the presence of another drug. 

**Figure 5:**
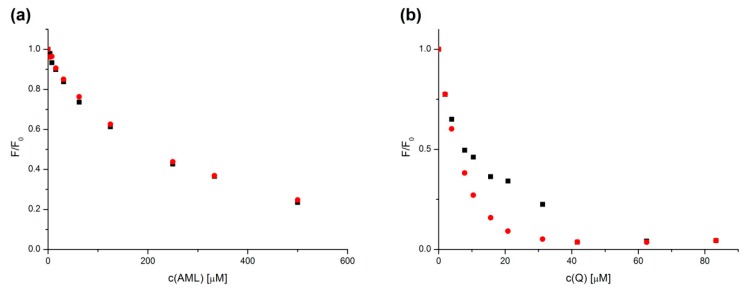
Quenching curves of HSA with AML (**a**) and with Q (**b**), respectively, for binary (black squares) and ternary (red dots) systems. Conditions: T = 310.15 K, pH = 7.4. The HSA concentration was 5 μM; AML and Q concentrations increased from 0 to 500 μM and from 0 to 83.3 μM, respectively.

Figure 5 shows the quenching curves comparison of binary and ternary systems. In the case of the AML binding, there was no discernible difference between the binary and the ternary systems where HSA was preincubated with Q. This means that the presence or absence of Q did not influence the ability of AML to bind to HSA. However, Figure 5b shows a case of a cooperative binding. When AML is bound first, Q causes stronger fluorescence quenching. This could be explained by allosteric modulation: the presence of AML inside the Sudlow’s Site 1 deforms the protein molecule in a way that allows for better access and stronger binding of Q. 

**Figure 6:**
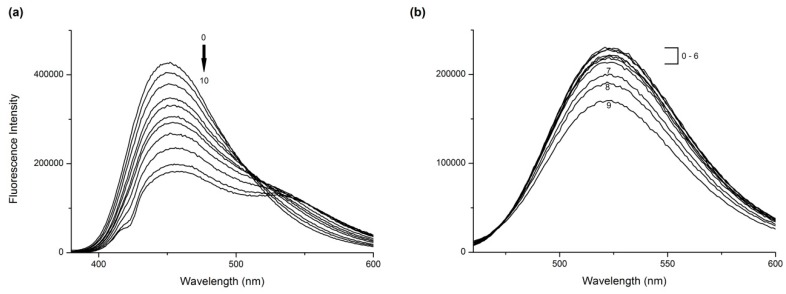
The fluorescence emission spectra of ternary systems (**a**) (AML + HSA) + Q at λ_EX_ = 370 nm and (**b**) (Q + HSA) + AML at λ_EX_ = 450 nm. Conditions: T = 310.15 K, pH = 7.4. The concentration of HSA and firstly-added drug was 5 μM; Q and AML concentrations increased from 0 (line 0) to 83.3 μM (line 10) and from 0 (line 0) to 500 μM (line 9), respectively.

In order to investigate if any displacement interaction was present, it was necessary to also observe the behavior of the firstly-added drug in ternary mixtures. Figure 6 shows the behavior of the fluorescence enhancement bands (characteristic for ligand + HSA) of the firstly added ligand in ternary mixtures. Upon addition of AML into Q + HSA system, we could see the band for Q + HSA sample stays almost intact and there was only a slight displacement at the highest AML concentrations. On the contrary, upon addition of Q into AML + HSA system, the enhanced fluorescence band of AML + HSA gradually decreased and that at the highest Q concentrations it was completely replaced by the characteristic double fluorescence band of Q + HSA system as seen in Figure 3. Therefore we can assume that Q was able to displace AML from its binding site, but in the case when Q was bound first it was not displaced and both drugs were binding at the same time to Sudlow’s Site 1.

### 2.2. Stern–Volmer Analysis

Fluorescence quenching can be described by the well-known Stern–Volmer equation:

F_0_/F = 1 + K_q_ [Q]

where F_0_ and F are the fluorescence intensities before and after a quencher addition, K_q_ is the Stern–Volmer quenching constant, and [Q] is the quencher’s concentration [23]. 

**Figure 7:**
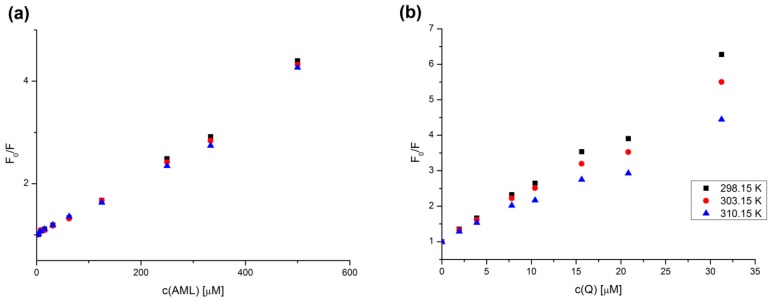
Stern–Volmer plots for (**a**) AML + HSA and (**b**) Q + HSA systems at different temperatures and λ_EX_ = 295 nm. The HSA concentration was 5 μM; AML and Q concentrations increased from 0 to 500 μM and from 0 to 31.25 μM, respectively.

It is possible to determine the type of fluorescence quenching by obtaining the K_q_ value at various temperatures. For AML the difference between Stern–Volmer plots at different temperatures is almost nonexistent and the plots display a slight upward curvature, concave towards the y-axis (Figure 7a). This is characteristic for the combination of static and dynamic quenching and the decrease of K_q_ with the increasing temperature is compensated by the increase of K_S_ (an association constant of static quenching). However, in high molar ratios, such as in this experiment, the upward curvature is rather caused by the extreme concentrations of the quencher molecule. As the quencher concentration increases, the probability increases that a quencher is within the first solvent shell of the fluorophore at the moment of excitation. These closely spaced fluorophore–quencher pairs are immediately quenched upon excitation, and thus appear to be dark complexes [23]. Still, from the fact that the K_q_ value stays the same for all temperatures, we can conclude the quenching mechanism is a combination of static and dynamic quenching. 

Figure 7b shows that for Q the K_q_ value significantly decreases with the increasing temperature, therefore the type of fluorescence quenching present is the static one characteristic by a fluorophore–ligand complex formation. This is in an agreement with the fluorescence enhancement experiments where we observed a specific emission band for Q + HSA ground-state complex. Q + HSA system similarly suffered from an extreme upward curvature at the highest concentrations (data not shown). Therefore, for the K_q_ calculations we used the first seven samples from each dataset starting from the lowest quencher concentration to avoid the distortion caused by extreme concentrations. 

From K_q_ we were able to determine the accessibility of fluorophores for quenching using the following equation.

*K*_q_ = k_q_ × τ_0_

where k_q_ is the bimolecular quenching constant and τ_0_ is the average fluorescence lifetime of the fluorophore without quencher (10^−8^ s for Trp) [23]. Table 1 is listing the K_q_ and k_q_ values for all binary and ternary systems at 310.15 K. The K_q_ values further support the behavior described by Figure 5. Values of k_q_ are larger than the maximum scattering collision quenching constant k_q_ (2.0 × 10^10^ L mol^−1^ s^−1^) for all systems, however the values for AML binding in both secondary and ternary systems are one order of magnitude lower than those of Q which might support the combination of static and dynamic quenching. 

### 2.3. UV Absorption Measurements

Careful examination of the absorption spectra of the fluorophore is an additional method to distinguish static and dynamic quenching. Collisional quenching affects the excited states of the fluorophores only and thus no changes in the absorption spectra are expected. In contrast, ground-state complex formation will frequently result in perturbation of the absorption spectrum of the fluorophore [23]. 

**Figure 8:**
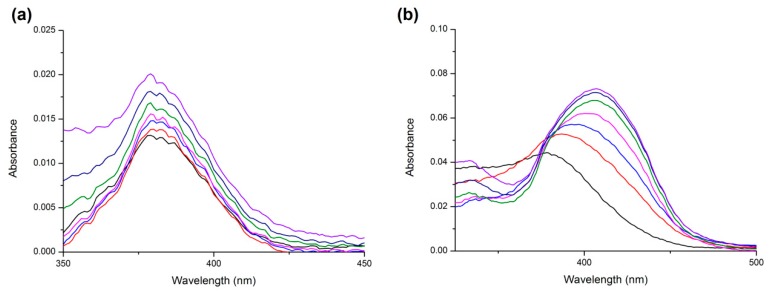
The UV absorption spectra of ligand + HSA systems for (**a**) AML and (**b**) Q. In all experiments, ligand concentration was 7.5 μM and HSA concentrations were 0 (black line), 1.87 (red line), 3.75 (blue line), 7.5 (pink line), 15 (green line), 20 (dark blue line), and 30 (purple line) μM. Conditions: T = 310.15 K, pH = 7.4.

In order to investigate this possibility, we performed UV absorption measurements of the systems from Figure 3 and focused on ligand absorption. In Figure 8 we see that the AML absorption band undergoes some minor changes in the intensity and no changes in the band shape. On the contrary, the UV absorption spectra of Q changes dramatically, with changes in the shape and the band maximum shifts from 375 nm (pure quercetin) to 407 nm (Q + HSA ground-state complex). These findings further confirm the results of Stern–Volmer analysis: Q forms a ground-state complex with Trp^214^ and AML quenches the HSA fluorescence by combination of static and dynamic quenching.

### 2.4. Binding Constant Analysis

Analysis of binding constants and thermodynamic parameters plays an important role in pharmacokinetic research. According to Klotz [24], widely used linear expression of fluorescence data (i.e., Scatchard plot) is not suitable for obtaining both the K_D_ value and the number of binding sites from a single dataset since in order to do that, at least two different measurements have to be performed. Moreover, all linear transformations of binding data suffer from similar limitations: minor nonlinearities are very difficult to detect and evaluate, point-spread masks the inaccuracies caused by titration errors, but most importantly, all linear transformations of binding data invalidate the rules for applying a linear transformation to a dataset. 

Lissi et al. [25] also confirmed that values for n (“number of binding sites”) obtained from the Scatchard plot were significantly different from values obtained by ultracentrifugation experiments. Suspiciously, all values of n from several dozens of published research articles are very close to one and much smaller than values obtained by ultracentrifugation experiments. There is also a common discrepancy in the actual meaning of n in this equation—where most research articles label it as “number of binding sites”, it in fact represents the stoichiometry of the binding step. 

Therefore we used a more simplistic approach by using the DynaFit software script. Data input consisted of [L_total_] (total ligand concentrations) as the variable reactant concentrations, [P_total_] (total protein concentration) as the fixed reactant concentration, and F/F_0_ as the experimentally observed value. The software creates a semilogarithmic plot of F/F_0_ vs [L_free_] (free ligand concentration) and determines the K_D_ value by finding the halfway point in the sigmoid-shaped curve. 

The results are displayed in Table 1. We can see that AML binding is two factors of ten weaker than that of Q. This result supports the observations that Q forms a ground-state complex and is able to displace AML from its binding site. From the comparison of K_D_ values for binary and ternary mixtures we can (in correlation with Figure 5) similarly conclude that the presence of Q does not change the binding affinity of AML and the presence of AML significantly increased the binding affinity of Q. 

From K_D_ values we were also able to calculate the values of ΔG (Gibbs free energy) using the following equation.

∆G = −RT ln (c^θ^/K_D_)

In which, R is the ideal gas constant, T the temperature, and the standard reference concentration c^θ^ = 1 mol/L. All ΔG values for all systems were negative. Therefore, it can be inferred that the binding interaction of HSA with all systems is spontaneous and enthalpy driven. 

### 2.5. Circular Dichroism Measurements

Circular dichroism spectroscopy is a valuable technique for detecting changes in the protein secondary and tertiary structure. In order to obtain an insight into the HSA structure, the far-UV (200–260 nm) and the near-UV (250–340 nm) CD spectra were recorded in the presence or absence of drugs in six molar ratios of ligands to protein (L/P) (0, 0.5, 1, 2, 3, and 4) for both binary and ternary systems. 

**Figure 9:**
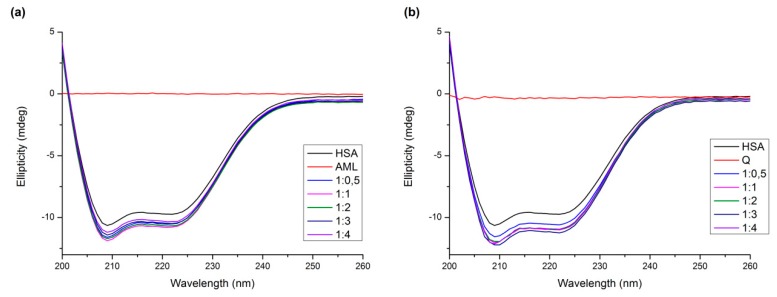
Far-UV CD spectra of (**a**) AML + HSA and (**b**) Q + HSA systems. Conditions: T = 310.15 K, pH = 7.4. The HSA concentration was 1 μM; AML or Q concentrations increased from 0 to 4 μM.

The far-UV CD spectrum of HSA showed two negative minima in the UV region at 208 nm and 222 nm, which is characteristic of the α-helical structure of the protein. The spectral profiles of the binary systems shown in Figure 9 indicate that the binding of Q or AML induced a minor perturbation in the HSA secondary structure. Table 2 lists the α-helix percentage values for all molar ratios. We can see that upon binding of AML, the protein reaches the maximum α-helix content at molar ratio 1:1 and then decreases again. The sample with Q, similarly, reaches maximum α-helix content at molar ratio 1:1 and the value continues to fluctuate around the similar percentage upon adding more Q into the solution. 

**Figure 10:**
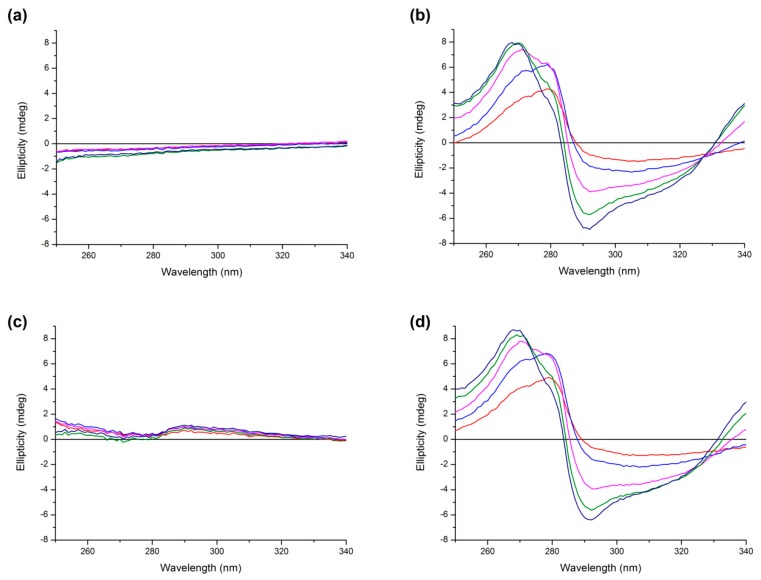
Near-UV CD spectra of (**a**) AML + HSA, (**b**) Q + HSA, (**c**) (Q + HSA) + AML, and (**d**) (AML + HSA) + Q. Conditions: T = 310.15 K, pH = 7.4. The HSA concentration was 15 μM while the concentrations of AML or Q were 7.5 μM (red line), 15 μM (blue line), 30 μM (pink line), 45 μM (green line), and 60 μM (dark blue line).

The near-UV CD spectrum is a useful tool for observation of the protein tertiary structure. Figure 10 shows the spectra for all binary and ternary systems. We can see that AML caused minimal changes in the CD spectrum. On the contrary, we see that upon addition of Q, the spectrum of HSA shows major perturbations of the tertiary structure, both in Tyr and Trp range (275–287 nm and 285–305 nm, respectively) [26] which is in agreement with the study by Zsila et al. [27]. In ternary mixtures we can see that upon addition of AML into Q + HSA system there was a small interaction in the Trp range which supports the claim that in the presence of Q, AML binds to a slightly different binding site near Trp^214^. In the vice versa situation, preincubation with AML caused a small decrease of quercetin’s Trp-range CD band and a small increase of Tyr-range CD band. 

### 2.6. FT-IR

**Figure 11:**
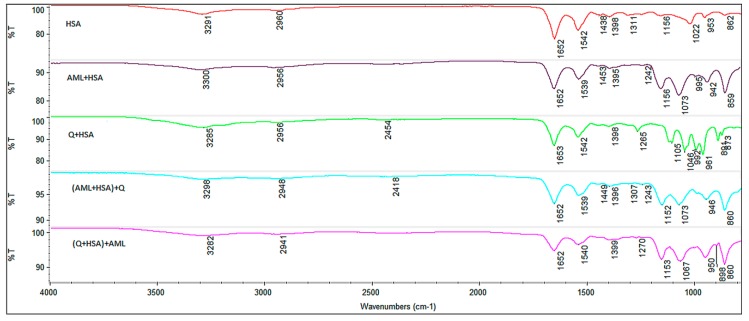
FT-IR spectra of HSA (red), AML+HSA (violet), Q+HSA (green), (AML + HSA) + Q (aquamarine), and (Q + HSA) + AML (magenta).

The secondary structure of HSA and its dynamics can be effectively studied by Fourier transform infrared spectroscopy. All proteins, including HSA, possess the protein amide I band at 1650–1654 cm^−1^ (C=O stretching) and amide II band at 1540–1560 cm^−1^ (C–N stretch coupled with N–H bending mode), which are related to the protein secondary structure [28]. The changes of the amide I and amide II bands observed in the FT-IR spectra are related to the interaction of protein with the analyzed drug. As shown in Figure 11, amide I band maxima for all studied samples are almost identical. 

That means binding AML or Q did not result in any major change in the secondary structure of HSA. However, amide II bands of most ligand + HSA samples are slightly downshifted. This suggests slightly different binding site near Trp^214^ for AML and Q, respectively. This supports our conclusions based on the CD spectra analysis. Subtle changes in the local environment of both ligands could be confirmed by deconvoluted band I analysis of all samples (spectra not shown), too. In short, three main bands (in decreasing intensity of order, after rounding the wavelength values) for free HSA are at 1651 cm^−1^ (random coil), 1660 cm^−1^ (α-helix), and 1667 cm^−1^ (β-turn); for the binary system Q + HSA we found bands at 1652 cm^−1^ (α-helix), 1648 cm^−1^ (random coil), and 1656 cm^−1^ (α-helix); for the binary system AML + HSA bands exist at 1660 cm^−1^ (α-helix), 1651 cm^−1^ (random coil), and 1667 cm^−1^ (β-turn); for the ternary system (Q + HSA) + AML we found bands at 1652 cm^−1^ (random coil), 1656 cm^−1^ (α-helix), and 1661 cm^−1^ (β-turn); and for the ternary system (AML + HSA) + Q we found bands at 1652 cm^−1^ (random coil), 1657 cm^−1^ (α-helix), and 1661 cm^−1^ (β-turn), respectively [29]. This confirms slightly different binding locations of quercetin and amlodipine in Site I of HSA as proposed by our CD spectra analysis and docking results.

### 2.7. Molecular Docking Study

**Figure 12:**
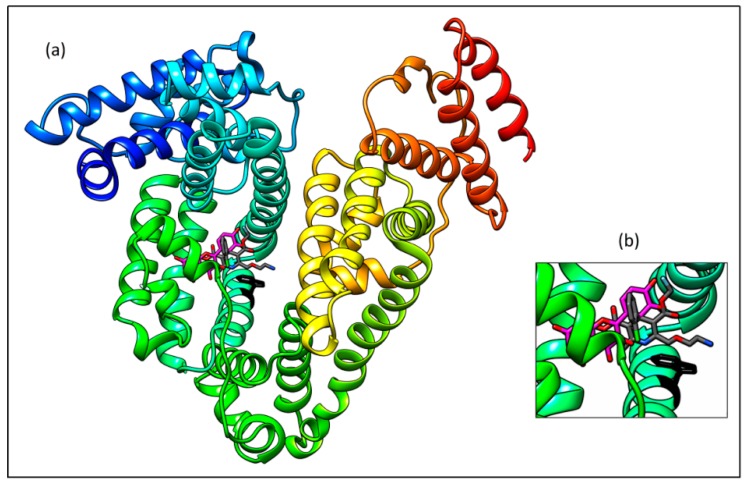
Superposition of the best docking placement of quercetin (magenta) and amlodipine (grey) in site I of HSA (**a**) and a detailed view (**b**); always in the proximity of Trp^214^ (black).

Both ligands (Q and AML) were separately docked and the same binding Site I was found. However, some difference in concrete position was discovered as shown by a superposition of best docking results (Figure 12a,b). The space occupied by AML is defined by the following amino acids; Tyr^150^, Glu^153^, Ser^192^, Lys^195^, Lys^199^, Trp^214^, Arg^222^, Leu^238^, His^242^, and Glu^292^. For Q, amino acids Tyr^150^, Ser^192^, Lys^195^, Lys^199^, Trp^214^, Arg^222^, Leu^238^, Leu^260^, and Ala^291^ define its placement on HSA. 

**Figure 13:**
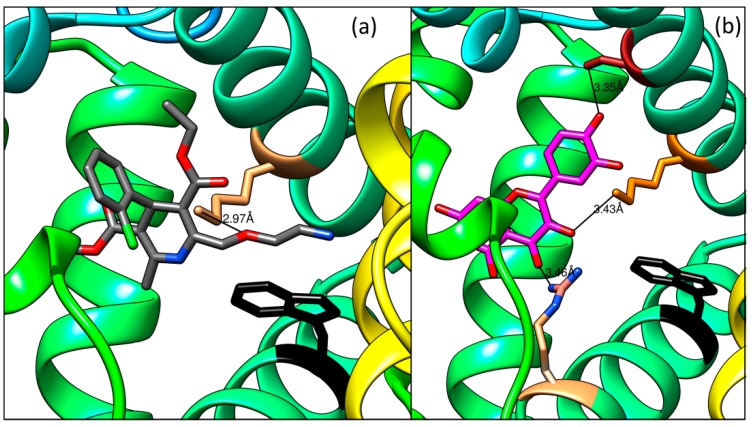
Detailed view on hydrogen bonds between (**a**) AML and Lys^199^ or (**b**) Q and Ser^192^, Lys^199^ and Arg^222^, respectively, in HSA.

Different positioning of both ligands in binding Site I is fixed by different hydrogen bonds: AML interacts through the oxygen (bearing an ethylamino group) with the amino group of Lys^199^; the length of this hydrogen bond is 2.97 Å (Figure 13a). For Q, three hydrogen bonds were found (Figure 13b) between

- the carbonyl oxygen and the amino group of guanidine part of Arg^222^ (distance 3.46 Å),
- the hydroxyl group on the C-3 and the amino group of Lys^199^ (3.43 Å), and
- the hydroxyl group on the C-4′ and the hydroxyl group of Ser^192^ (3.35 Å)

These results support the interpretation of all our spectroscopic measurements.

## 3. Discussion

Our results demonstrate for the first time, to the best of our knowledge, the binding interactions between quercetin and amlodipine on human serum albumin. From the results shown above it can be concluded that both drugs bind to the same primary binding site localized inside the Sudlow’s Site 1 on HSA and there exist a competitive interaction between them. Quercetin is binding with the higher affinity and is able to displace amlodipine from its binding site. Amlodipine binds with the lower affinity and if the binding site is already occupied by quercetin, it binds with the same affinity to the secondary binding site inside the same hydrophobic pocket of Sudlow’s Site 1. The displacement of amlodipine by quercetin may elevate the concentration of the unbound amlodipine in the serum which might cause fluctuations in patient’s blood pressure or elevated risk of adverse side effects. However, more studies, particularly in vivo monitoring of the free plasma levels of drugs, should be performed to evaluate the magnitude and severity of this interaction.

These results help further the knowledge of binding interactions and are helpful for understanding the interactions between plant compounds and drugs. In concurrence with other studies mentioned in this work, the plant compound–drug interactions are known to cause adverse side effects and therefore should be treated with similar caution as any other drug–drug interaction.

## 4. Materials and Methods

### 4.1. Materials

Human serum albumin (recombinant, expressed in rice), amlodipine besylate (pharmaceutical secondary standard), quercetin (≥95%, HPLC), and dimethylsulfoxide (DMSO; ≥99.5% (GC) plant cell culture tested) were purchased from Sigma-Aldrich. Phosphate buffer (50 mM, pH 7.4) was prepared from Na_2_HPO_4_ × 12 H_2_O and NaH_2_PO_4_ × 2 H_2_O (p.a., Centralchem, Slovakia). The HSA stock solutions were prepared by dissolving an appropriate amount in phosphate buffer. AML stock solutions were prepared by mixing an appropriate amount of the substance with phosphate buffer and then heating the mixture to approx. 60 °C until completely dissolved. Quercetin stock solutions were prepared by dissolving the substance in DMSO and then diluting in phosphate buffer to the required concentration. DMSO concentration in final mixtures did not exceed 1% (v/v). Milli-Q water was used for all the measurements.

### 4.2. Methods

#### 4.2.1. Fluorescence Measurements

Fluorescence spectra were measured in triplicates on a FluoroMax 4 spectrofluorimeter (Horiba Jobin Yvon Scientific, Edison, NJ, USA), equipped with a 1.0 cm path length quartz cell. The slit widths for the excitation and emission were 3.0 nm for all measurements. The temperatures used for the measurements were 298.15 K, 303.15 K, and 310.15 K, respectively. Samples were incubated for 2 min. Buffer background was subtracted from the raw spectra. Fluorescence intensities were corrected for the absorption of excitation light and reabsorption of emitted light to decrease the inner filter using the following relationship [30]:

$$F_{cor}=F_{obs}\times 10^{(A_{ex}+A_{em)/2}}$$

where *F_cor_* and *F_obs_* are the corrected and observed fluorescence intensities, respectively. *A_ex_* and *A_em_* are the absorbance values at excitation and emission wavelengths, respectively.

#### 4.2.2. Binding Constant Analysis

Fluorescence spectral data after correction were used to calculate the dissociation constant (K_D_) and Gibbs free energy (ΔG) for all studied systems. The 10 nm wide section around the fluorescence maximum was selected and the fluorescence intensity values were added up to minimize the influence of signal noise.

For evaluation and logarithmic plot fitting we used DynaFit software (DynaFit 4; BioKin, Ltd.: Watertown, MA, USA, 2015) using a custom-written script.

#### 4.2.3. UV Absorption Measurements

UV absorption spectra were performed on Infinite M200 Tecan (Männedorf, Switzerland) using Sarstedt TC Plate 96 Well, Standard, F. The temperature was 310.15 K. Plates were incubated for 2 min. Buffer background was subtracted from the raw spectra.

#### 4.2.4. Circular Dichroism (CD) Measurements

The isothermal wavelength scan studies of HSA in the absence or the presence of Q and/or AML were carried out in triplicates using a Chirascan CD spectrophotometer equipped with a Peltier type temperature controller (Applied Photophysics Ltd., Leatherhead, UK). The instrument was flushed with nitrogen with a flow rate of 5 L per minute, the path length was 1 mm, spectral bandwidth was set to 1 nm, the scan time per point to 5 s, and the temperature was set to 310.15 K. Buffer background was subtracted from the raw spectra.

For the far-ultraviolet (far-UV) CD spectra (200–260 nm) the HSA concentration was 1 μM. For the near-UV CD (250–340 nm) spectra an HSA concentration of 15 μM was used. Six molar ratios of ligands to protein (L/P) (0, 0.5, 1, 2, 3, and 4) were investigated for both binary and ternary systems.

The far-UV spectral data were used to calculate the α-helix percentage using the following equation [31].

$$\alpha \;helix\;\%=\frac{-MRE_{208\;\mathrm{nm}}-MRE_{\beta \text{-}sheets}}{-MRE_{\alpha \text{-}helix}-MRE_{\beta \text{-}sheets}}\times 100$$

where *MRE*_208 nm_ is the mean residue ellipticity value of the sample at the excitation wavelength of 208 nm, *MRE_α-helix_* is the standardized value for a protein with 100% content of *α-helixes* and is equal to 33,000, and *MRE_β-sheets_* is the standardized value for a protein with 100% content of *β-sheets* and is equal to 4000.

The near-UV spectral data were evaluated by an empiric method described by Zsila et al. [26,32] The spectra were brought down to a common baseline by subtracting the spectrum of pure HSA.

#### 4.2.5. FT-IR

For this method, purified lyophilized crystallic samples were prepared. The concentrations of HSA and ligands for the FT-IR spectra analyses were 10 μM and 80 μM, respectively. The solutions were mixed and incubated for 15 min, then filtered through 30 kDa ultracentrifugation filters Amicon^®^ Ultra 0.5 mL using Hettich Universal 320 laboratory centrifuge. The filtrate was freeze-dried and used as a FT-IR sample.

ATR-FT-IR spectra of all samples were recorded on a Nicolet 6700 FTIR spectrometer. All spectra were taken on a germanium crystal with a resolution of 4 cm^−1^ and using 32 scans at 298 K. The number, position and width of component bands were estimated by performing a Fourier self-deconvolution to the protein infrared amide I band after subtraction of the free HSA spectrum from the sample ones using Omnic 9 software (Thermo Fisher Scientific Inc.: Waltham, MA, USA). The featureless original HSA spectrum between 2200 and 1800 cm^−1^ was the subtraction criterion.

#### 4.2.6. Docking Study

Human serum albumin structure from the Protein Data Bank (PDB ID: 1E78A) was used for calculations. Quercetin and amlodipine structures were created in ChemSketch software (ChemSketch, version 12.01, Advanced Chemistry Development, Inc., Toronto, ON, Canada) and converted from a *.mol file format to a *.pdb one by OpenBabel 2.3.2 and used without any optimization. To be in line with conditions in fluorescence and CD experiments (pH = 7.4) a corresponding protonized amlodipine molecule (based on ACD/ADME Suite version 5, Build 1339 prediction; Advanced Chemistry Development, Inc.: Toronto, ON, Canada) was used for docking. A PatchDock web server (http://bioinfo3d.cs.tau.ac.il/PatchDock/index.html) was used to dock (complex type: protein–small ligand, clustering RMSD = 4.0). Best 10 docking solutions were evaluated for both ligands. Molecular graphics images were produced using the UCSF Chimera 1.13 package (Resource for Biocomputing, Visualization, and Informatics at the University of California, San Francisco, CA, USA). Hydrogen bonds were calculated using this package using relax constraints of 0.4 Å and 20.0 degrees, respectively.

## Figures and Tables

**Table 1:** The values of Stern–Volmer quenching constants (K_q_), bimolecular quenching rate constants (k_q_), correlation coefficients of Stern–Volmer curves (R), binding constants (K_D_), and Gibbs free energy (ΔG) for the binary and ternary systems at λ_EX_ = 295 nm and T = 310.15 K.

System	K_q_ [10^3^ M^−1^]	k_q_ [10^11^ M^−1^s^−1^]	R	K_D_ [μM]	ΔG [kJ mol^−1^]
AML + HSA	5.35 ± 0.09	5.35 ± 0.09	0.9998	183.77 ± 25.28	−22.181
Q + HSA	15.23 ± 1.89	15.23 ± 1.89	0.9756	6.48 ± 2.01	−30.807
(Q +HSA) +AML	5.07 ± 0.07	5.07 ± 0.07	0.9998	192.81 ± 24.09	−22.057
(AML + HSA) + Q	76.02 ± 1.35	76.02 ± 1.35	0.9989	2.39 ± 0.32	−33.379

**Table 2:** The values of α-helix percentages for binary systems AML + HSA and Q + HSA.

System	Ratio Protein:Ligand	α-Helix Content [%]
AML + HSA	1:0	59.1 ± 1.0
	1:0.5	63.6 ± 0.3
	1:1	64.7 ± 0.2
	1:2	64.1 ± 0.3
	1:3	62.5 ± 0.1
	1:4	61.5 ± 0.7
Q + HSA	1:0	59.1 ± 1.0
	1:0.5	63.0 ± 2.0
	1:1	65.4 ± 0.5
	1:2	65.2 ± 0.9
	1:3	66.2 ± 0.7
	1:4	66.3 ± 1.5
